# Linking climate change, global mental health and structural interventions: opportunities for research and action

**DOI:** 10.1192/bjo.2026.11002

**Published:** 2026-05-11

**Authors:** Siqi Xue, Sean A. Kidd, Muhammad Ishrat Husain, Nusrat Husain, Charlotte Hanlon

**Affiliations:** Centre for Addiction and Mental Health (CAMH), Toronto, Ontario, Canada; Department of Psychiatry, Temerty Faculty of Medicine, University of Toronto, Toronto, Ontario, Canada; Krembil Brain Institute, University Health Network (UHN), Toronto, Ontario, Canada; Division of Psychology and Mental Health, University of Manchester, Manchester, UK; Global Centre for Research on Mental Health Inequalities, Mersey Care NHS Foundation Trust, Prescot, UK; Division of Psychiatry, Institute for Neuroscience and Cardiovascular Research, University of Edinburgh, Edinburgh, UK; Department of Psychiatry, School of Medicine, College of Health Sciences, Addis Ababa University, Addis Ababa, Ethiopia

**Keywords:** Climate change, social determinants of mental health, structural interventions, global mental health, natural disasters

## Abstract

Climate change disproportionately affects people with pre-existing mental illness, yet there is a critical shortage of targeted interventions serving their needs. This Commentary argues for the further development and evaluation of preventative, structural interventions, including cash transfers, in the context of climate-change related disasters to reduce vulnerability among people with mental illness.

**Figure:**
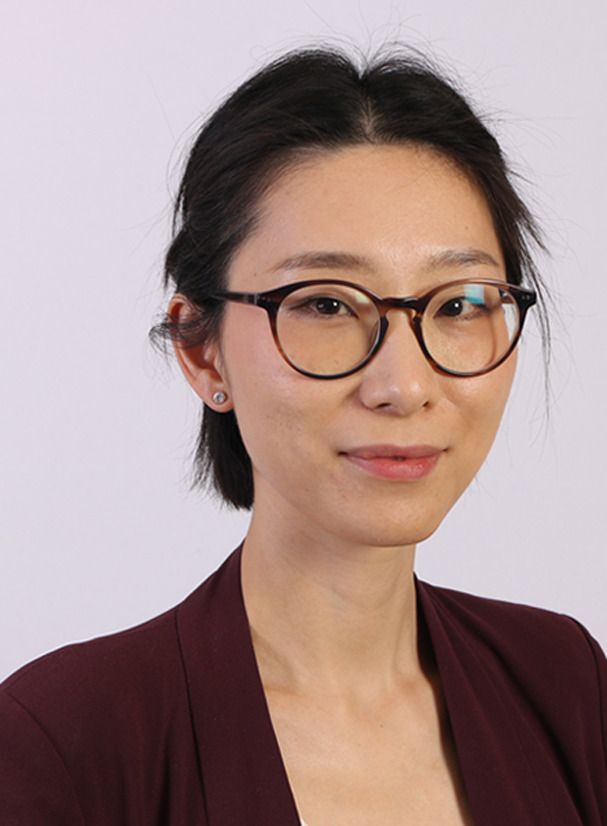

## Response

Climate change is increasingly recognised and incorporated into frameworks of the determinants of mental health.^1,2^ A growing body of research demonstrates the association between climate change and a range of adverse mental health outcomes, including the exacerbation of pre-existing mental illness.^3^

People with mental illness have consistently been identified as an at-risk group that experiences disproportionate morbidity and mortality during climate change-related extreme weather events.^4^ Nevertheless, to date, few climate-adaptation and disaster-preparedness programmes explicitly consider people with mental illness. A recent review of heat-health action plans worldwide revealed a critical gap of actionable interventions for people with mental illness, especially in low- and middle-income countries (LMICs).^5^ This clear misalignment of known risks and absence of targeted interventions reflects structural discrimination, whereby the needs of people with mental illness are systematically deprioritised.

To better safeguard people with mental illness and their well-being in the context of climate change, we argue that designing and evaluating structural interventions is essential. Existing interventions at this intersection mostly target emotional responses, change behaviours or treat symptoms.^6^ Although these are important components of care, they are insufficient to address the milieux that shape climate vulnerabilities, including poverty, poor housing and discrimination, that too often affect people with mental illness. Structural interventions, by contrast, are those that modify these social and environmental conditions rather than an individual’s clinical conditions.

Calls to reform global mental health increasingly emphasise the need to rebalance towards preventative and population-level strategies.^7^ Inclusive, structural interventions represent an opportunity to address both the global mental health burden and associated social determinants to reduce vulnerabilities to climate-related hazards. In this Commentary, we focus on poverty as a structural determinant and priority target for intervention.

Poverty is modifiable and considered an upstream driver of other social determinants for people with mental illness. Furthermore, in the current conceptual model connecting climate change with mental health outcomes, poverty is identified as a key mediator.^8^ Climate hazards damage homes, destroy livelihoods and lead to forced relocations, with the financial stress leading to psychological distress at both the individual and household level. People with mental illness often lack the economic and social buffers to withstand these shocks.

Poverty alleviation through cash transfers is among the most well-studied forms of structural interventions. Although these have increasingly been used to support community climate resilience, mental health outcomes are rarely measured.^9,10^ Recent evidence also highlights opportunities to integrate poverty-reduction with psychological interventions to strengthen positive mental health impacts.^11^ However, their effectiveness in the context of climate change-related events remains understudied.^12^ Several studies build the evidence base that cash transfers could offer the dual benefits of supporting climate resilience and mental well-being. In a Red Cross Red Crescent project in Bangladesh, forecast-based cash transfers in anticipation of severe floods were associated with reduced stress and anxiety symptoms among low-income households.^13^ In Ethiopia, pastoral communities that received a capacity-building intervention that included a donor grant component reported better overall health and more optimism after experiencing a drought.^14^

However, there is a lack of preventative, structural interventions designed for people with mental illness, particularly those residing in weather-susceptible locations in LMICs. To date, cash-based and poverty-reduction interventions in climate-affected settings have largely been implemented at the level of the general population or low-income households, and may even systematically exclude people with mental illness based on stigma. As a result, the implications, feasibility and mental health impacts of such interventions for this population remain largely unknown. People with mental illness represent a distinct dimension of vulnerability in the context of climate change, beyond poverty, given their higher psychosocial needs, greater barriers to service access and reduced adaptive capacity. Structural interventions such as cash transfers may plausibly support their well-being through multiple pathways. These pathways include meeting basic survival needs, improving financial stability, enhancing self-reliance and agency and facilitating continuity of care and social participation during and after extreme weather events.

Operationalising such interventions targeting people with mental illness would require integration into existing vulnerability and social protection systems.^15^ Mental illness diagnosis could be incorporated into multisectoral vulnerability assessments that consider other relevant factors, including household-level poverty, the presence of young children and/or older dependants in the household, single-woman-headed households and chronic physical disease or disability, to ensure that the most climate-vulnerable are prioritised.

In addition to directly addressing poverty, opportunities exist to target other structural determinants of mental health in the context of climate change. Housing is a particularly urgent issue.^16^ People with mental illness are disproportionately affected by poor-quality housing or homelessness, which contributes to excess mortality related to heatwaves.^17^ Cash transfers may enable recipients to retrofit their homes to be more resilient to extreme weather events, and to better afford cooling devices and energy expenses, including generators in the event of an electricity outage. Gender-based violence is another dimension of vulnerability in the context of climate change. Women with severe mental illness are more likely to experience sexual and domestic violence, the incidence of which increases with extreme weather events.^18,19^ Because cash-transfer programmes have been shown to decrease intimate partner violence, there is potential for these, along with emergency shelter planning for women, to be integrated into mental health and climate-response strategies.^20^ These examples illustrate how structural interventions can operate across multiple determinants, such as income, housing and safety, to reduce compounded climate vulnerabilities.

Future research is needed to clarify causal pathways linking climate hazards with adverse mental health outcomes, especially for people living with severe mental illness in LMICs, where intersecting risks are most pronounced. Further development of theoretical frameworks (e.g. specifying which subgroups are most vulnerable and when interventions should be delivered) would be important in supporting the design of more targeted and testable interventions. Additionally, future economic evaluations should assess whether structural interventions implemented in climate-affected settings reduce downstream costs to health systems.

At the policy level, mental health should be explicitly integrated into national climate-adaptation plans and disaster risk-management frameworks, rather than being addressed through the health sector alone. Coordination across ministries of health, social welfare, housing, gender and climate/environment is required to identify shared vulnerability criteria. The multisectoral response may be supported by financing mechanisms, such as the expansion of existing cash-transfer programmes to account for mental health-related vulnerability.

In conclusion, climate change underscores the need to anchor mental health interventions within a social justice lens. Structural interventions, such as cash transfers, should be coupled with clinical care in climate-response strategies to support people with mental illness and their long-term resilience. By targeting upstream determinants, the global mental health community can better align with the development, human rights and climate-resilience agendas. Achieving this will require the deliberate integration of mental health considerations into social protection and climate-adaptation plans, alongside a clearer articulation of mechanisms through which structural interventions influence mental health outcomes. The development and evaluation of, and investment in, structural interventions for mental health will be essential for building equitable mental health care programmes in a changing climate.
